# Improvisational science

**DOI:** 10.1186/s13059-021-02575-w

**Published:** 2022-01-03

**Authors:** Itai Yanai, Martin Lercher

**Affiliations:** 1 Institute for Computational Medicine, NYU Langone Health, New York, NY 10016 USA; 2 Institute for Computer Science & Department of Biology, Heinrich Heine University, 40225 Düsseldorf, Germany

The fun is always on the other side of a ‘yes’.

Martin DeMatt (improvisational theatre instructor)

To a first approximation, scientific research is composed of two distinct domains. The executive domain is concerned with the testing of hypotheses in a robust and consistent manner. This is the one governed by the scientific method that is taught from elementary schools to universities. Its complement is the creative side of science, which generates the initial insights and ideas that lead to the hypotheses in the first place. The two domains are intimately linked in what we have called the data-hypothesis conversation [1]. Following François Jacob [2], we have called these two domains day science and night science [3]. While scientific progress relies equally on advances in both domains, the methods and practices of night science are not well understood, and perhaps for this reason are not formally taught.

To better understand night science, we have been writing on its use of analogies, the discovery of new questions by embracing contradictions, hypotheses as a liability, and importing/exporting ideas across fields [3–8]. Beyond these aspects centered on individual scientists, night science creativity also has an important social component. Indeed, one of the factors most conducive to scientific creativity is having someone that you can talk to; the development of most scientific projects can be cast as one long discussion between a small set of people. Through those discussions, it becomes possible to improvise new ideas that eventually become the material of day science testing. So while day science is premised on accuracy and planning, night science revels in improvisation, often between a pair of scientists.

In preparation for this editorial, we had done our research. The question was then how to get it onto the page. In the spirit of improvisational science—and its relation in the arts, improvisational theater—we found it appropriate to record a spontaneous conversation on these topics among the authors, edited only lightly for clarity and supplemented with references where appropriate.

**Itai:** You know – we live in a world that believes in the myth of the lone genius: someone working in their office and having their best ideas alone. But really there’s just something undeniably powerful about being able to talk through your ideas with someone. Somehow that really moves the thinking forward [9].

**Martin:** I think that’s an experience that most scientists go through: you can have ideas all by yourself, but a crucial part of the process is talking to a person you trust and who understands your ideas.

**Itai:** Right, trust is important here, because many of us – maybe all of us – constantly feel like we’re imposters. Having someone you can share your half-baked ideas with means that you can feel comfortable enough to make yourself vulnerable.

**Martin:** I think most ideas, while you’re trying to find them, are much worse than only half-baked: a lot of my initial ideas are genuinely stupid.

**Itai:** Exactly. Most of your ideas are stupid! Just kidding. But one in a thousand of those stupid ideas will actually be the seed of something good, and you’ll never know if you don’t express them all.

**Martin:** Yes. If you don’t feel comfortable sharing your worst idea, it’s going to be very hard to find your best one [10].

**Itai:** Right, and what I like about talking with some of my favorite collaborators is that they can actually do the ‘yes, and’ principle.

**Martin:** The what?

**Itai:** It’s when I’m saying something, and the person I’m talking with – instead of changing the topic – instead of shooting it down – instead of saying “No, but…” – actually goes along with it. And tries to build on it.

**Martin:** That sounds a lot like improvisational theater, doesn’t it?

**Itai:** I like to think of it like that. The players are working together, each making their own contribution. But the goal is not to stick to any preformed story, but to generate an entirely new story. It’s kind of like Jazz too in that sense, where the goal is not to stick to the tune but to transcend it. So, when you're listening to John Coltrane play 'My Favorite Things', you hear him explore what is possible within the confines of the tune [11].

**Martin:** ‘Improvisational Science’ then.

**Itai:** Exactly. To be creative, you have to open up and improvise, also in science.

**Martin:** There is a difference between improvisational theatre and science, though. In improvisational theater, I could at any point invoke a gorilla swinging across the stage – my only limit is the previous development of tonight’s improvised play. But in science, things eventually need to be consistent with all the knowledge already out there.

**Itai:** You’re absolutely right. But there is a time and place for everything. I would say that the improvisational part governs the realm of night science – where you’re trying to make connections and form ideas. Here you’re not so much trying to provoke a reaction from an audience, of course – but it’s improvisational in the sense that you don’t know where the conversation is going ahead of time. There is no plan. Then to get to the consistency that you’re talking about, you use day science, which is just a totally different realm of science where the formal scientific method applies.

**Martin:** But if we’re discussing some unexplained phenomenon, I can’t just agree with everything you say. It would be like evolution with only mutations and no selection. You know, you might think of generating ideas as a kind of random, mutational process, and of distinguishing the promising ideas from the not-so-promising ones as natural selection. Is it efficient to always wait for day science before we check something against what we know?

**Itai:** I see your point and I think that it’s a delicate balance. The aspect, I think, that goes a bit unappreciated is that when we are talking, we should try to make the other person look good. If instead we just shoot down everything they say – even if we were just correctly stating the truths as we see them – then we don’t give the other person a chance to develop something that eventually could be interesting.

**Martin:** That reminds me of a Jazz story. Miles Davis played a concert with Herbie Hancock. Hancock mistakenly played the wrong chord. A complete harmonic disaster. He was in horror. But instead of continuing a melodic line that would have made Hancock’s error obvious, Miles Davis paused for a moment and then proceeded to play notes that harmonized with Hancock’s chord – he turned the mistake into something interesting, musically [12].

**Itai:** I love that story, because it’s the same in improvisational science: if one person says something apparently stupid, then it’s the job of the other person to help them with it.

**Martin:** I think now we’re converging on something we can agree on. The “Yes, and…” rule is maybe not to be taken too literally. It’s a spirit, a frame of mind: be positive about anything the other person says, rather than rejecting it, see if you can together reshape it to make it better.

**Itai:** Right. It’s an attitude. And it seems to be almost like a lost art. There is such an emphasis now on efficiency and on being a fast thinker. But it’s super important to remember that when you’re having these conversations, you need to suspend all of that. You need to believe that some slow thinking and just going around and around the same old topics may somehow uncover a crack in the wall that you didn’t notice before.

**Martin:** I think if you tried to do that by yourself, you’d run the risk of going in circles. And then there is nothing new - you never get away from where you already are. But if you walk with another person, you may be much more likely to go in unexpected directions. To continue the mutation/selection analogy: I think that exchanging ideas with another person is like recombination, accelerating the evolution of new ideas by forming new combinations across people.

**Itai:** Right. And talking with another person also motivates us to actually spend time thinking deeply. When I’m just sitting alone, I feel too much pressure to use my time to reply to some emails and it sort of feels too indulgent – or maybe I’m not disciplined enough – to just sit there and think. But if I schedule time to meet with another person, then it just happens that there is this space to actually think. It’s as though we have a social contract and we feel legitimate in thinking and doing night science together. It’s like going to the gym with another person: you show up because you don’t want to let them down. If it were just you, you’d just as easily press the snooze button.

**Martin:** I like the idea of a social contract: I’m at my most creative when I sit together with a PhD student, discussing new approaches or new analyses. Because I know they need creativity to make progress in their projects, and I know it is my job to help them with this.

**Itai:** Yes, and I used to call these meetings ‘brainstorming’. But now I think that we should call that process ‘improvising’. Brainstorming suggests that we should invite a bunch of people in for an idea generation session, where we write down lots of ideas and then choose those we like best. This is good for some purposes maybe. But it doesn’t really capture the way I like to talk science with my collaborators.

**Martin:** I like that new term. From now on, I’ll be inviting my collaborators for “a coffee and some improvisation”!

**Itai:** I’m assuming that you’re not planning to invite them all together. I know that some people think that with these things it is ‘the more the merrier’, and they set up meetings with a large group. That can be done when it’s a group that really has developed a lot of trust. For example in my lab’s group meeting, I think we are able to speak openly and creatively; but that is a very special group that has worked hard to develop that kind of trust. Ad-hoc groups don’t have that. And in those settings, people tend to behave very differently – they feel stifled, for example.

**Martin:** There is even research that shows that larger groups excel at developing an existing field: papers with many authors tend to get many citations. But the real novelties that disrupt science are more likely to come from the smallest groups, often involving only two people. So this suggests that large groups are efficient, but they’re not that creative [13].

**Itai:** And it’s interesting that you mentioned an improvisation between you and your student. I do think that improvisational science works best when it’s with just one other person. And social psychologists have shown that we really do behave differently depending on our group size. The group-think mentality – the influences of peer pressure – are very strong [14]. To get real feedback on an idea, it’s just incredibly useful to talk to just one other person – or at least one at a time.

**Martin:** True, one can think of so many creative pairs, across disciplines: Watson/Crick, Lennon/McCartney, Brown/Goldstein, Christo/Jean-Claude, Kahnemann/Tversky. I’m particularly impressed by the creativity of the last pair. Kahnemann and Tversky together developed a new perspective on human decision making - coming up with their hypotheses during long walks, where they talked about their personal experiences in decision making. You need to feel very comfortable with talking to a person to have the openness required for this kind of talking [15].

**Itai:** And from the outside, if you were just looking at these two people going on their long walks, you might have thought that they were not actually working. But really, they were doing the toughest part of their work. They were thinking about an idea that excited them for some reason – a reason they might not have been able to define initially. And they imagined experiments and thought through what might work and what might not. It’s as though the executive part of science comes only after you’ve thought through everything. And there is no better way to think through something than with one other person.

**Martin:** Why not ten other persons? If you just want to think something through?

**Itai:** Ten would be great - if it’s one after the other in small groups. But all together? Imagine someone saying something that in a small setting could start a whole discussion, but in a group of ten, one person could just shoot it down. And that would be the end of that idea.

**Martin:** So on the one hand, the social aspects of talking are crucial. But on the other, improvising doesn’t work as well when there are too many people around. I think there are at least two other aspects why talking boosts our creativity. The first one is that we need help to question our favorite assumptions. As Harmit Malik puts it: you may have explained your model of some process a hundred times to different people, when suddenly one person asks you about a trivial step: how do you know that? And you realize that you cannot really answer that question – maybe there was a wrong assumption here that had misled your thinking all the time [16].

**Itai:** Talking with another person has a way of jolting us out of our routine thinking. It’s like we get locked into driving around endlessly in a traffic rotary when another person could - from their perspective very easily - point out an exit to a new place.

**Martin:** It may not even present itself as an exit, it could look more like a roadblock - but then, by finding a way around it, an exit may emerge. There’s another and much simpler aspect of why talking helps, though: explaining our ideas to someone else forces us to transform a fleeting network of thoughts in our brain into a linear, logical structure.

**Itai:** And then, as we say our thoughts in this linear structure, we often realize it’s actually not all that logical and the flaws of the argument jump out at us.

**Martin:** In principle, a blackboard or a written document could serve the same purpose - but apparently, based on how our brains are wired, that doesn’t appear to be the optimal setting. Maybe because in evolution, humans more frequently encountered other humans than blackboards or documents.

**Itai:** That’s interesting that you say that. I was just thinking when you started talking about blackboards that probably for a mathematician a lot of the thinking does happen in isolation and that the blackboard provides the benefit that we talked about. But you’re right, humans are social beings, and our brains are optimized for social interaction. So what about the mathematicians?

**Martin:** The mathematicians may actually be the most collaborative of all scientists. Famously, the elevator at the Isaac Newton Institute for Mathematical Sciences in Cambridge has a blackboard - not for the lone mathematicians to further develop their ideas on the way to lunch, but for quick discussions of strange new ideas with random colleagues [17].

**Itai:** I love it. And indeed, new ideas do sound very strange when you first hear them, almost by definition. It reminds me of Plato’s analogy of the cave, where the person who has received a glimpse of some new aspect of reality is treated by their peers as an outcast [18]. The reason that talking about a strange new idea in a small group helps is that it sort of eliminates the politics, the peer pressure to conform, and other nonsense, and allows the idea to develop.

**Martin:** And it’s even worse when you’re still on the way to find that new idea. It’s crucial to be able to let your mind drift in a somewhat random, unpredictable fashion. In a way, maybe night science is like a randomized, genetic optimization algorithm: you’re trying to find the best explanation, but there is no predetermined way that leads you there. Day science, in contrast, may be more like a gradient descent – it will get you to an optimum much more quickly, but you may end up in a local optimum rather than finding the global optimum you were looking for.

**Itai:** It’s just clear that to get moving with a project, you need to find someone that you can talk to. And if the person you need to discuss a specific aspect is not around, there is a great approximation you can use: you just have an imaginary conversation with that person in your head. Tzachi Pilpel claims this is how he got some of his best ideas – maybe this trick allows him to fool his brain into using its social interaction superpowers [19].

**Martin:** But probably nothing beats a real conversation. It may also be that Tzachi is referring to the period in between discussions. After all, the notion of the lone genius is not a complete myth; there really is a need for periods of intense work and those typically happen in isolation. It’s just that periods of improvisational science with a colleague are juxtaposed in between these.

**Itai:** It’s almost paradoxical. These improvisational conversations are all around us - out in the open - and yet they may be the hidden part of the scientific method.

Our goal for this conversation was to convey the importance of a particular kind of conversation in science for generating ideas. We call it “improvisational” rather than brainstorming because of its emphasis on (1) small group size, ideally just a pair of people; (2) exercising the “yes, and” principle common to other forms of improvisation; and (3) an emphasis on a relationship and an atmosphere of trust—“making the other person look good.” Like a muscle, our improvisational science skills require constant exercise to work their magic—as we improvise more with other scientists, our creative potential grows.
